# Combinational therapy targeting the MET‐mTOR‐ROS loop disrupts mitochondrial autoregulatory machinery of liver cancer

**DOI:** 10.1002/ctm2.237

**Published:** 2020-12-01

**Authors:** Xing Huang, Gang Zhang, Xueli Bai, Tingbo Liang

**Affiliations:** ^1^ Zhejiang Provincial Key Laboratory of Pancreatic Disease the First Affiliated Hospital, School of Medicine Zhejiang University Hangzhou China; ^2^ Department of Hepatobiliary and Pancreatic Surgery the First Affiliated Hospital, School of Medicine Zhejiang University Hangzhou China; ^3^ Innovation Center for the Study of Pancreatic Diseases Hangzhou China

**Keywords:** checkpoint, combination therapy, MET, mTOR, ROS

## Abstract

A wide variety of regulators have been identified in mechanistic target of rapamycin (mTOR) activation; however, the protective mechanisms of mTOR inactivation are still largely unknown, especially in tumor growth. Here, we have found the hepatocyte growth factor (HGF) receptor (MET) is required for mTOR activation‐stimulated mitochondrial oxidative phosphorylation (OXPHOS) in a phosphorylation‐dependent manner in liver cancer. Intriguingly, we observed mitochondrial quality dictates the regulatory effects of MET on mTOR and OXPHOS. Once overloaded, mitochondrial reactive oxygen species (ROS) inhibits mTOR activity and OXPHOS performance to prevent mitochondrial dysfunction‐induced tumor cell death, by disrupting MET dimerization to block its autophosphorylation and interaction with vacuolar ATP synthase (V‐ATPase). The MET‐mTOR‐ROS loop acts as a protective checkpoint in liver cancer, and thus this autoregulatory machinery is a promising combinational target for liver cancer therapy.

**Abbreviations**
AA: amino acids
HGF: hepatocyte growth factor
KD: kinase‐dead
KO: knockout
mTOR: mechanistic target of rapamycin
MET: HGF receptor
NAC: *N*‐acetyl‐L‐cysteine
OXPHOS: oxidative phosphorylation
Redox: oxidation‐reduction reaction
ROS: reactive oxygen species
S6K1: ribosomal protein S6 kinase 1
TPR‐MET: translocated promoter region fused to MET
V‐ATPase: vacuolar ATP synthase
WT: wild‐type

Dear Editor,

Hyperactivation of mechanistic target of rapamycin (mTOR) signaling is frequently observed in cancer and aging.^1^, ^2^, ^3^ However, the cellular protective function of mTOR inactivation remains poorly characterized, which may explain the limited therapeutic efficacy of mTOR inhibitors in liver cancer.^4^ Accumulating evidence suggests systematic coordination between growth factor‐receptor axis and mTOR signaling.^5^, ^6^, ^7^, ^8^ Here, we focused on MET, a tyrosine kinase receptor for hepatocyte growth factor (HGF)^9^, ^10^ and a novel regulator of the mTOR machinery.^7^, ^11^

We observed amino acids (AA)‐enhanced phosphorylation of the ribosomal protein S6 kinase 1 (S6K1), a biomarker of mTOR activation, was repressed in MET knockout (KO) HepG2 cells. This phenomenon could be rescued by forced expression of wild‐type (WT) MET (Figure S1A). In support of this, the MET inhibitor Capmatinib exhibited near‐total inhibition of S6K1 phosphorylation (Figure S1B). Furthermore, we found reintroduction of a kinase‐dead (KD) MET mutant was unable to rescue MET KO‐caused mTOR inactivation (Figure S1C), while a constitutively active translocated promoter region fused to MET (TPR‐MET) mutant enhanced AA‐stimulated S6K1 phosphorylation (Figure S1D), suggesting MET mediates AA–mTOR signaling pathway in a phosphorylation‐dependent manner.

Interestingly, AA‐stimulated mitochondrial oxidative phosphorylation (OXPHOS), including oxidation‐reduction reaction (Figures S2A and B) and ATP production (Figures S2C and D), was severely compromised in MET KO cells. Parallel impairments were observed in mice with liver‐specific MET deficiency (Met^Liver‐KO^) (Figures S2E and F). We subsequently found HGF greatly enhanced AA‐stimulated OXPHOS (Figures S2G and H), and only WT MET could restore functional defects induced by MET KO (Figures S2I and J). Using the vacuolar ATP synthase (V‐ATPase) inhibitor Concanamycin A and the mTOR inhibitor Rapamycin, we further demonstrated effects of MET on OXPHOS were dependent on the V‐ATPase–mTOR axis (Figure S3A‐D).

Intriguingly, we observed the mitochondrial respiratory chain‐targeting inhibitor Metformin, or Oligomycin A plus Antimycin A, dramatically inhibited dimerization of MET (Figures S4A and B) and its phosphorylation (Figures S4C and D). Consequently, OXPHOS dysfunction disrupted the MET–ATP6V1A interaction (Figures S4E and F) and AA‐stimulated mTOR activation (Figures S4G and H). Both HGF and TPR‐MET exacerbated OXPHOS dysfunction‐caused cell death, which could be largely rescued by the inhibition of V‐ATPase or mTOR (Figures S5A and B). Reconstitution assays in MET KO cells also showed only WT MET exacerbated cell death caused by dysfunctional OXPHOS (Figures S5C and D). Briefly, OXPHOS dysfunction propels a feedback to prevent persistent mTOR activation‐caused impairment.

Mechanistically, we found the reactive oxygen species (ROS) scavenger *N*‐acetyl‐L‐cysteine (NAC) efficiently restored the impairment of mTOR activation in a MET‐dependent manner (Figures 1A and B). NAC could also largely rescue MET dimer formation and its phosphorylation from OXPHOS dysfunction (Figures 1C and D). Moreover, WT MET could not rescue mTOR activity suppressed by OXPHOS dysfunction, unless simultaneously administered with NAC; TPR‐MET was the most efficient mTOR activator and was not affected by ROS, whereas the MET KD mutant totally lost such function (Figures 1E and F). Furthermore, we observed NAC rescued OXPHOS dysfunction in a MET‐dependent manner (Figures S6A and B). Hence, ROS inhibits mTOR activation through disrupting MET dimerization to maintain the balance between AA metabolism and OXPHOS.

**FIGURE 1:**
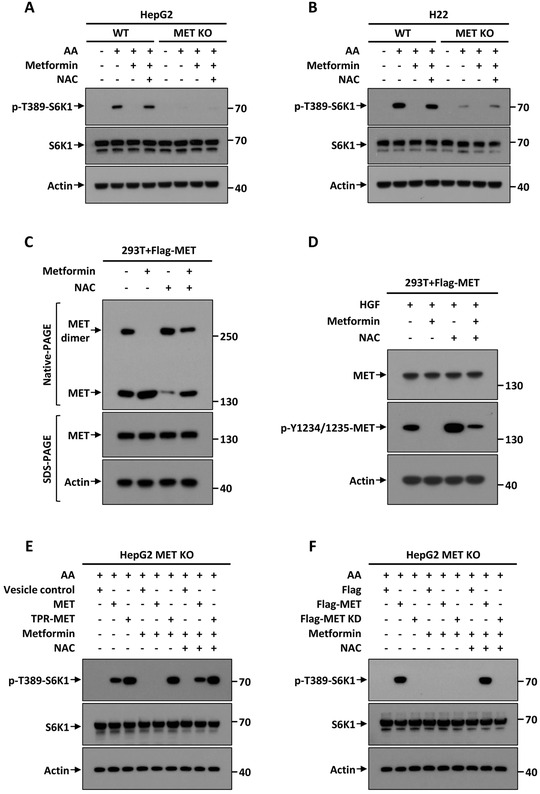
ROS generated from OXPHOS dysfunction suppresses mTOR activation by preventing MET dimerization and phosphorylation. A and B, ROS generated from OXPHOS dysfunction reverses AA‐stimulated mTOR activation via MET. WT and MET KO HepG2 (A) or H22 (B) cells (5 × 10^4^) were individually pre‐incubated with or without 2 mM metformin for 12 hours or/and 5 mM NAC for 4 hours, then starved for 90 minutes and subsequently stimulated with AA for 45 minutes. Cell lysates were analyzed by immunoblot. C, Mitochondrial ROS disrupts dimerization of MET. HEK‐293T cells expressing Flag‐MET (1 × 10^5^) were incubated with or without 2 mM metformin for 12 hours or/and 5 mM NAC for 4 hours, then subjected to Native‐PAGE and SDS‐PAGE, respectively and subsequently analyzed by immunoblot. D, mitochondrial ROS disrupts phosphorylation of MET. HEK‐293T cells expressing Flag‐MET (1 × 10^5^) were stimulated with HGF then incubated with or without 2 mM Metformin for 12 hours or/and 5 mM NAC for 4 hours. After treatment, cell lysates were analyzed by immunoblot. E, TPR‐MET fusion protein abrogates mitochondrial ROS‐induced mTOR inactivation. MET KO HepG2 cells (5 × 10^4^) were individually infected with WT MET, TPR‐MET, or vesicle control for 48 hours and were then treated with or without 2 mM metformin for 12 hours or/and 5 mM NAC for 4 hours. After treatment, cells were deprived of AA for 90 minutes then stimulated with AA for 45 minutes, and the resultant cell lysates were subjected to immunoblot analysis. F, MET‐KD mutant cannot rescue mitochondrial ROS‐induced mTOR inactivation. MET KO HepG2 cells (5 × 10^4^) were individually infected with Flag‐MET, KD mutant or vector control for 36 hours, and then treated with or without 2 mM metformin for 12 hours or/and 5 mM NAC for 4 hours. After treatment, cells were deprived of AA for 90 minutes then stimulated with AA for 45 minutes, and the resultant cell lysates were subjected to immunoblot analysis

Notably, overexpression of MET could promote AA‐stimulated mTOR activation even in autophagy deficient condition caused by genetic deletion of *Atg5* (Figure S7A), or chemical disruption of autophagy flux by Bafilomycin A1 (Figure S7B), which suggested autophagy has no influences on MET‐controlled mTOR activation. We further explored the impacts of autophagy on the ROS‐driven feedback loop, and results showed even under autophagy deficiency, MET still could facilitate AA‐stimulated mTOR activation (Figure S7C) and OXPHOS (Figure S7D); moreover, OXPHOS dysfunction‐caused ineffectiveness of MET regulation still could be rescued by NAC. This suggests autophagy is not involved in mitochondrial ROS suspending MET‐mTOR‐OXPHOS axis.

To investigate the pathological significance of MET‐mTOR‐ROS loop in tumor growth, we firstly analyzed the impacts of mTOR inhibition and ROS elimination on MET deficient cancer cells in vitro. WT and MET KO HepG2 cells were individually treated with NAC or NAC plus Rapamycin, and results indicated significant synergistic effects of MET and mTOR on the proliferation (Figure S8A), viability (Figure S8B), and colony formation (Figures S8C and D) of cancer cells under ROS clearance. The similar tendencies were also found in Capmatinib‐treated MET‐driven TPRMET‐NIH3T3 cell line (Figure S8E‐H).

To determine whether the MET‐mTOR‐ROS loop is a viable therapeutic target in liver cancer, we constructed multiple mouse models (Figure 2A). In accordance with our in vitro experiments, a combination of Capmatinib and Rapamycin exhibited the strongest inhibitory effects on the SMMC‐7721 xenograft model, while NAC reversed proliferative suppression caused by MET inhibition (Figures 2B and C). Similar findings were observed in the Huh‐7 xenograft model (Figures 2D and E) and the human TPR‐MET‐driven NIH3T3 model (Figures 2F and G). Furthermore, Kaplan‐Meier curves clearly demonstrated Capmatinib combined with Rapamycin significantly increased, whereas NAC remarkably reduced, the survival rates in both the H22 and Hepa1‐6 models (Figures 2H and I).

**FIGURE 2:**
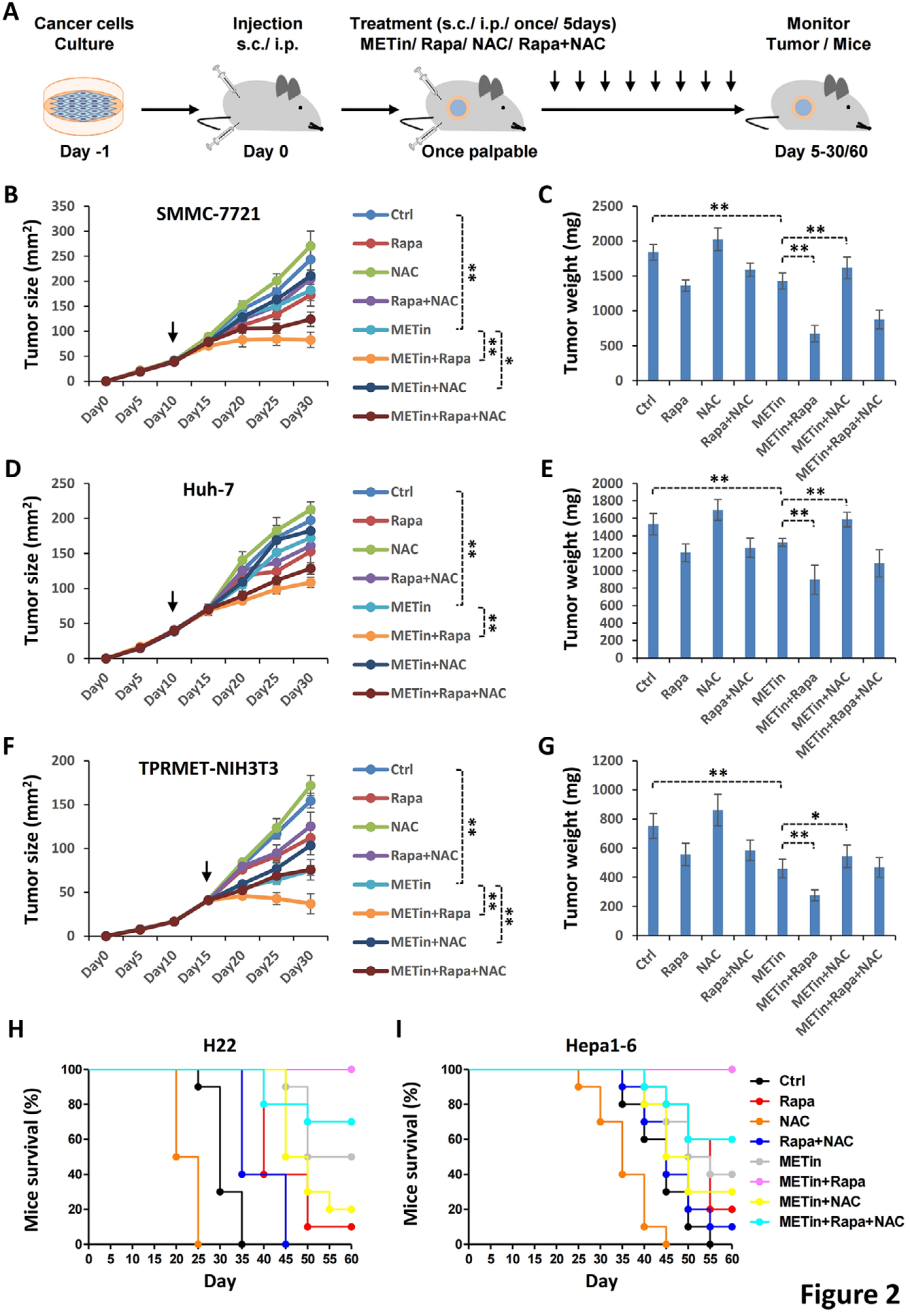
MTOR inhibition improves, and ROS elimination suppresses, MET‐targeted cancer therapy. A, Strategy for evaluating synergistic effects of MET‐mTOR inhibition and ROS elimination in MET‐targeted cancer therapy. B and C, MTOR inhibition improves, and ROS elimination suppresses the effects of MET inhibition on tumor growth in the SMMC‐7721 xenograft model. SMMC‐7721 cells (1 × 10^6^) were inoculated subcutaneously into the right flank of *nu*/*nu* mice. Once the tumor reached 37.5‐42.5 mm^2^, mice received vehicle control PBS (Ctrl, 100 μL), capmatinib (METin, 20 mg/kg), rapamycin (rapa) (10 mg/kg) or/and NAC (100 mg/kg), respectively. Treatments were administered by subcutaneous multi‐point injection adjacent to tumors every 5 days. Tumor growth was regularly reported as tumor size (B) and tumor weight (C). D and E, MTOR inhibition improves, and ROS elimination suppresses the effects of MET inhibition on tumor growth in the Huh‐7 xenograft model. Huh‐7 cells (1 × 10^6^) were inoculated and treated as described above, and tumor growth was regularly reported. F and G, MTOR inhibition improves, and ROS elimination suppresses the effects of MET inhibition on tumor growth in the TPR‐MET‐NIH3T3 xenograft model. TPR‐MET‐driven NIH3T3 cells (5 × 10^6^) were inoculated and treated as described above, and tumor growth was regularly reported. H and I, MTOR inhibition improves, and ROS elimination suppresses the effects of MET inhibition on the survival of the H22 and Hepa1‐6 ascite mouse models. H22 (H) and Hepa1‐6 (I) cells (5 × 10^5^) were individually inoculated intraperitoneally (i.p.) into *nu*/*nu* mice. After 10 days, mice were treated with PBS (Ctrl, 100 μL), METin (30 mg/kg), rapa (15 mg/kg), or/and NAC (150 mg/kg), respectively. Treatments were administered by i.p. injection every 5 days. The mortality was regularly reported with 10 mice per group over 2 months in a Kaplan‐Meier plot. Data are presented as the mean ± SD. Statistically significant differences using a two‐tailed Student's *t*‐test are marked as * (*P* < .05) or ** (*P* < .01)

Additionally, to evaluate the physiological role of the MET‐mTOR‐ROS loop in liver development, WT and Met^Liver‐KO^ mice individually received Rapamycin or/and NAC (Figure S9A). Results suggested Rapamycin significantly decreased, while NAC obviously increased, liver weight in Met^Liver‐KO^ mice (Figure S9B). Supporting this, the hepatocytes isolated from the mouse models showed consistent changes in cell viability (Figure S9C). Furthermore, WT and Met^Liver‐KO^ mice were used to investigate the impact of MET‐mTOR‐ROS loop on mice survival (Figure S9D). Results indicated the enormous role of Rapamycin in extending, and conversely NAC in reducing, the overall survival of Met^Liver‐KO^ mice (Figure S9E).

In conclusion, we have identified herein that MET as a central component of the mTOR sensing machinery in liver cancer. MET is required for AA‐stimulated mTOR activation and mitochondrial OXPHOS in a phosphorylation‐dependent manner. Mitochondrial quality in turn dictates MET phosphorylation status and its binding to V‐ATPase. Once overloaded, mitochondrial ROS inhibits mTOR activity and OXPHOS to avoid mitochondrial dysfunction‐induced tumor cell death, by targeting MET dimerization with disruption of its phosphorylation and interaction with V‐ATPase. We provide a schematic model to illustrate the MET‐dependent homeostatic maintenance between mTOR activation and OXPHOS (Figure S10A). Through integrating signals of AA and ROS, the MET‐mTOR‐ROS loop plays a protective checkpoint‐like role in liver cancer. We therefore propose this autoregulatory machinery is a promising combinational target for liver cancer therapy. Notably, the indeterminate influence of ROS^12^ and the potential side‐effects of mTOR inhibition^13^ should be further investigated in translation of such therapeutic strategies into clinical practice.

## CONFLICT OF INTEREST

The authors declare that there is no conflict of interest that could be perceived as prejudicing the impartiality of the research reported.

## AUTHOR CONTRIBUTIONS

Xing Huang, Xueli Bai, and Tingbo Liang conceived the project and supervised the study. Xing Huang designed and conducted the major experiments, Gang Zhang helped for metabolic assay, and all authors were involved in data analysis and interpretation. Xing Huang and Gang Zhang wrote and revised the manuscript, other authors discussed and commented on the manuscript, and all authors approved the final manuscript version. Xing Huang and Gang Zhang contributed equally to the drafting process. Xing Huang, Xueli Bai, and Tingbo Liang share senior authorship.

## ETHICS APPROVAL

The mice experiments performed in this study were approved by Ethnics Committee of Southeast University.

## CONSENT FOR PUBLICATION

All the authors consent for publication.

## Supporting information

Supporting Figure

Supporting Figure

Supporting Information

## Data Availability

All the data obtained and/or analyzed during the current study are available from the corresponding authors on reasonable request.
