# Supplementary figures and images for: Combinational therapy targeting the MET‐mTOR‐ROS loop disrupts mitochondrial autoregulatory machinery of liver cancer
Source: Clin Transl Med. 2020 Dec 1;10(8):e237. doi: 10.1002/ctm2.237 (PMC7708774; doi:10.1002/ctm2.237)

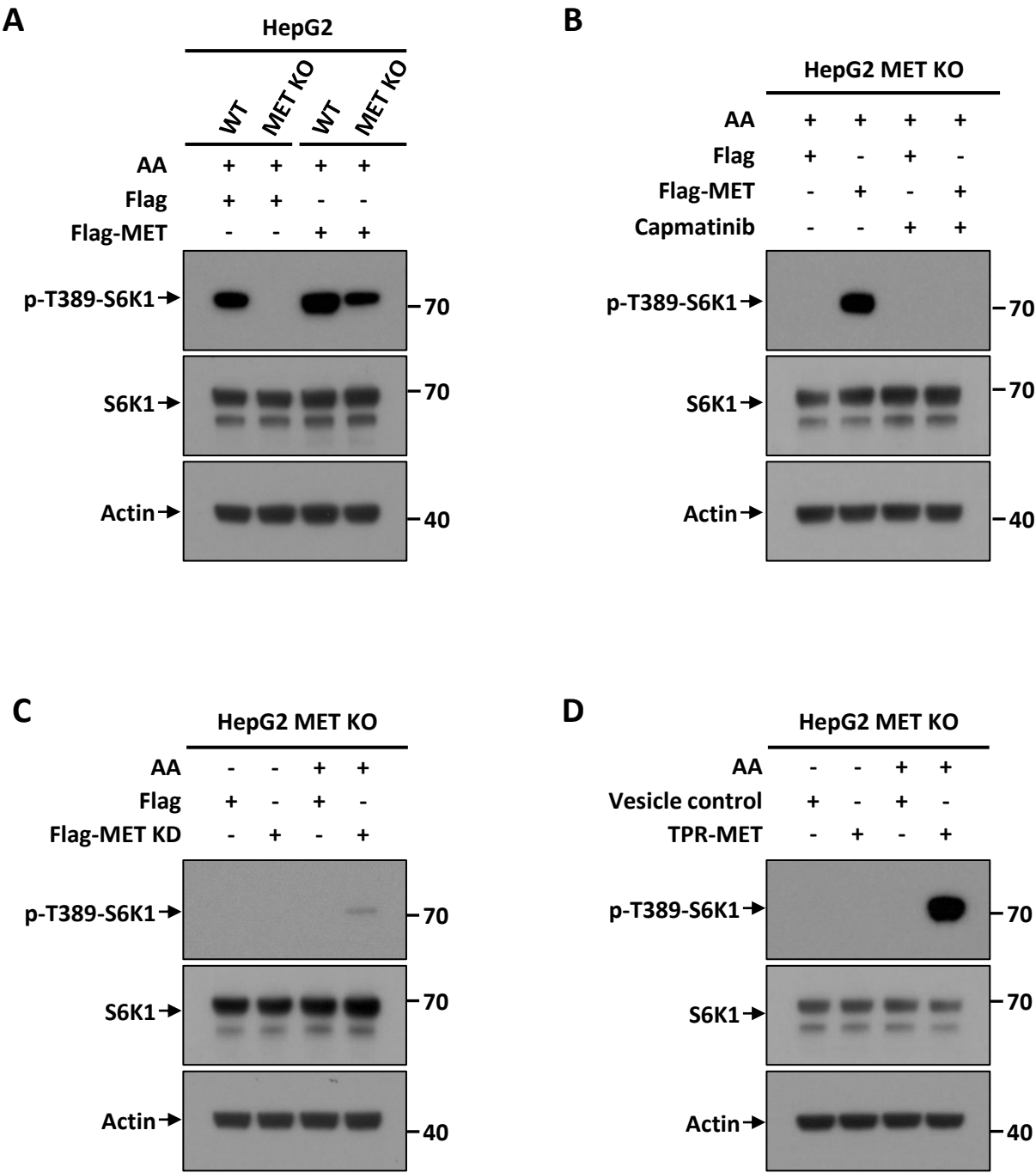

Figure S1

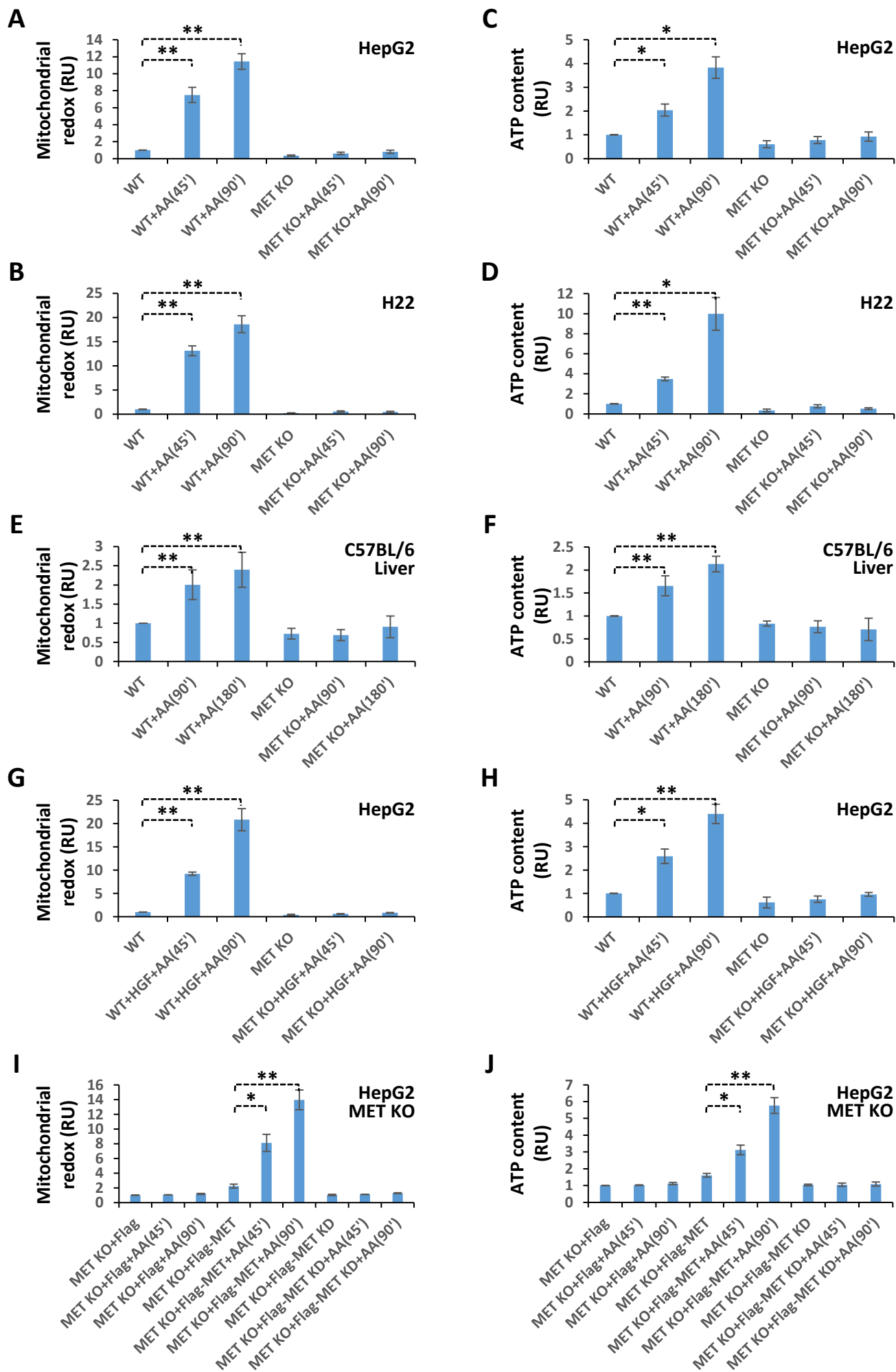

Figure S2

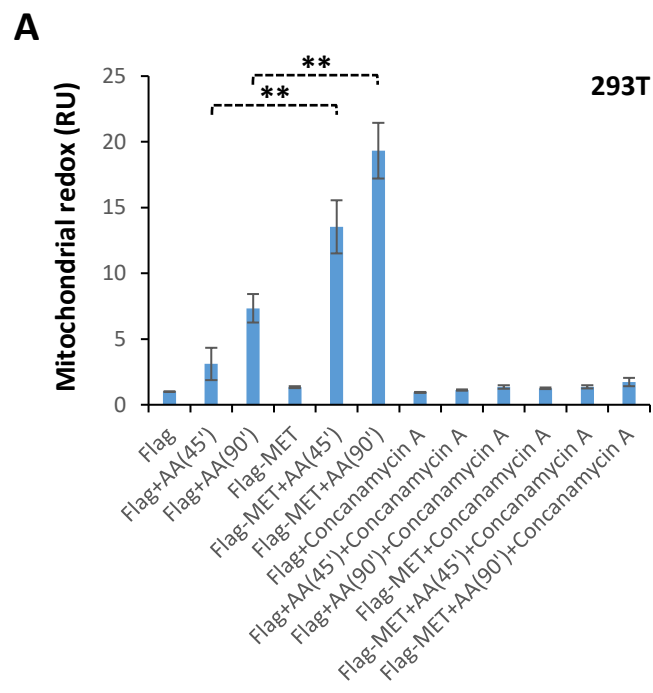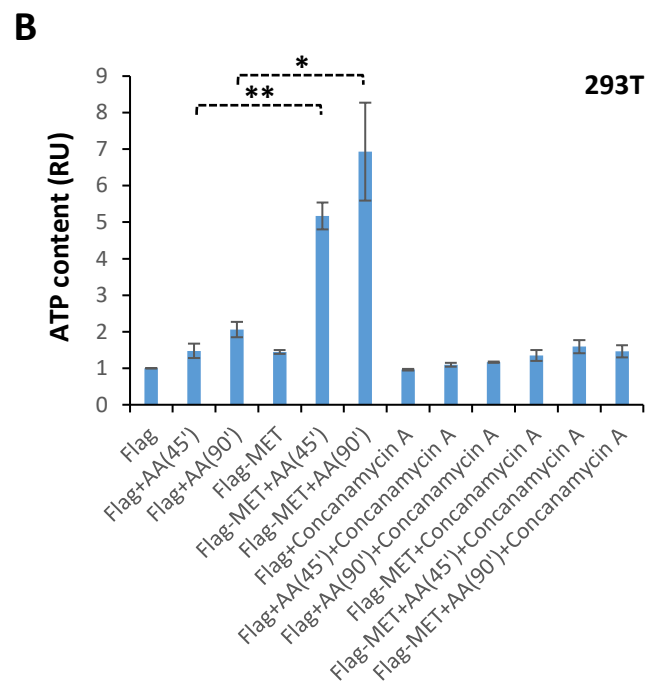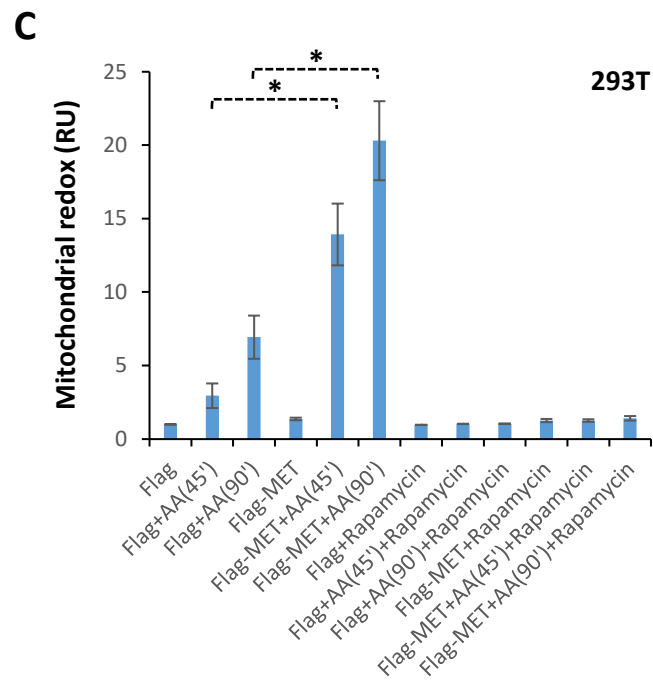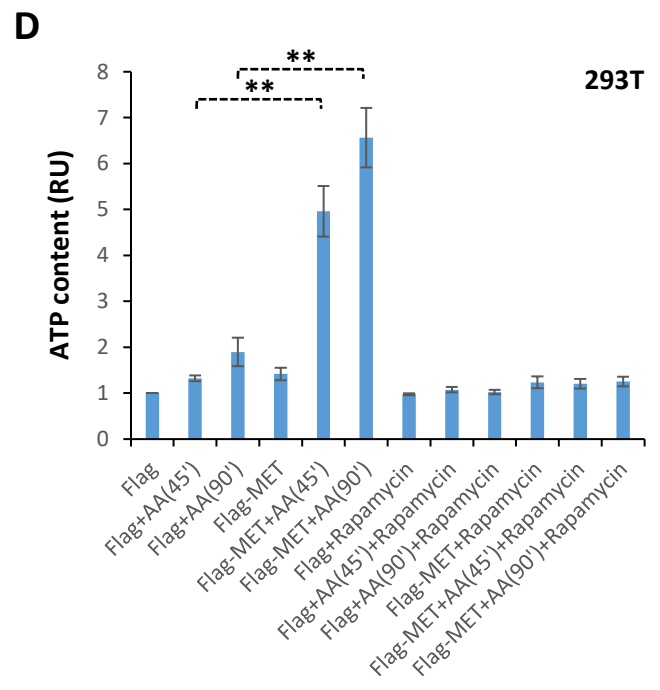

**Figure S3**

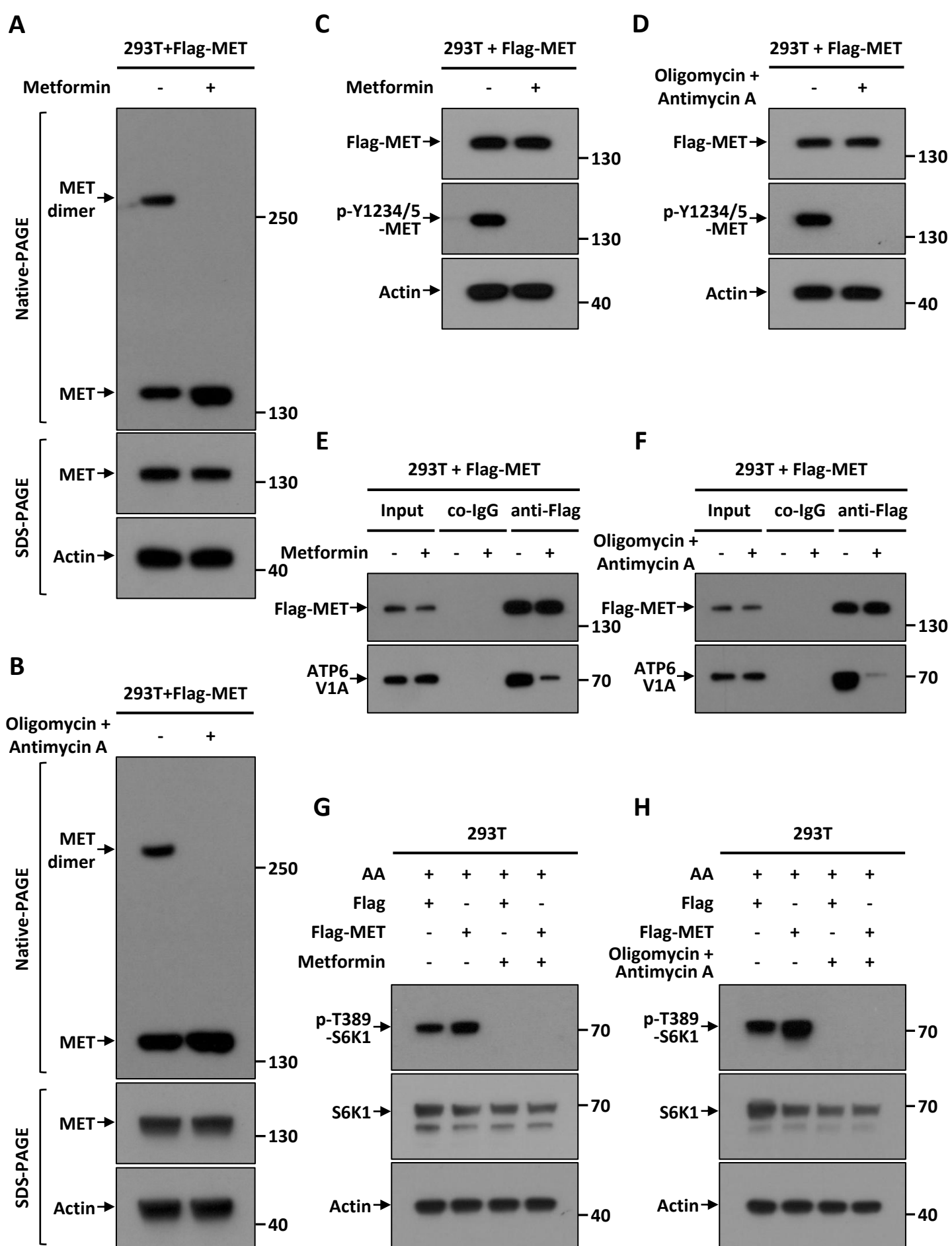

**Figure S4**

**A**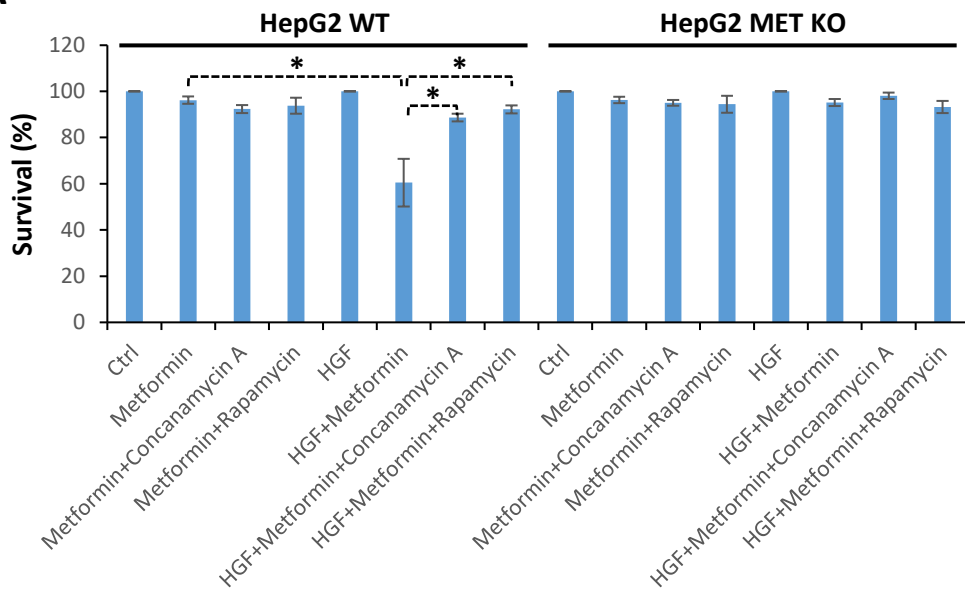**B**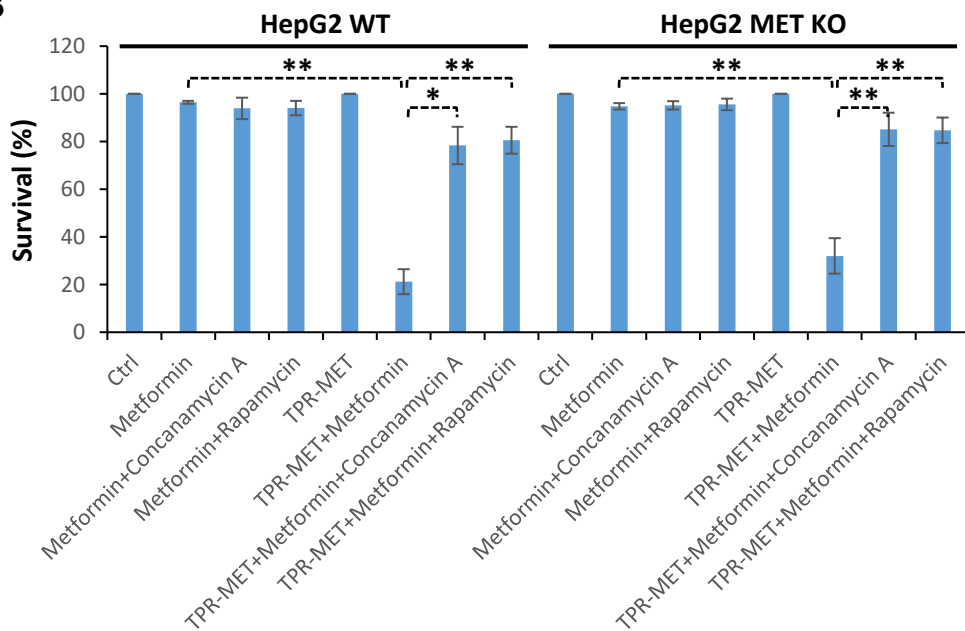**C**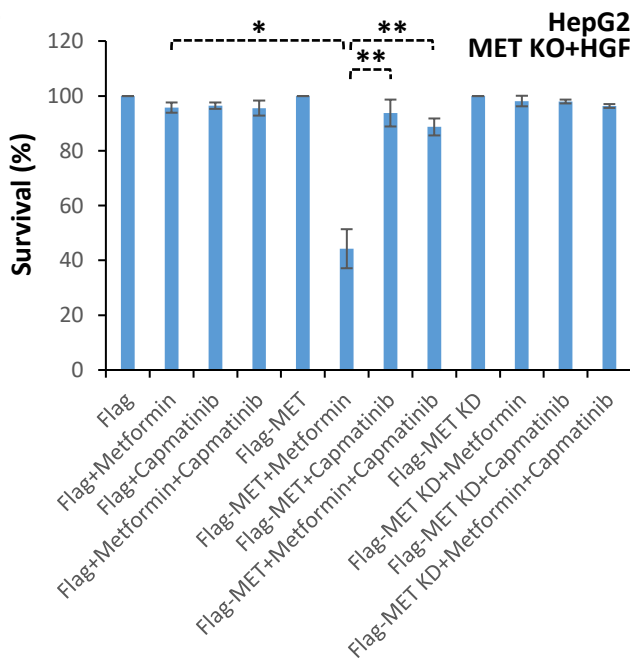**D**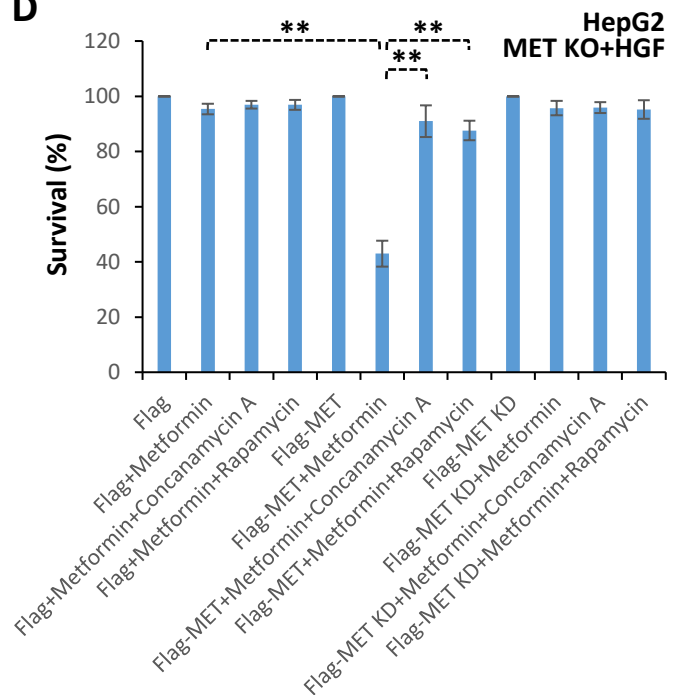**Figure S5**

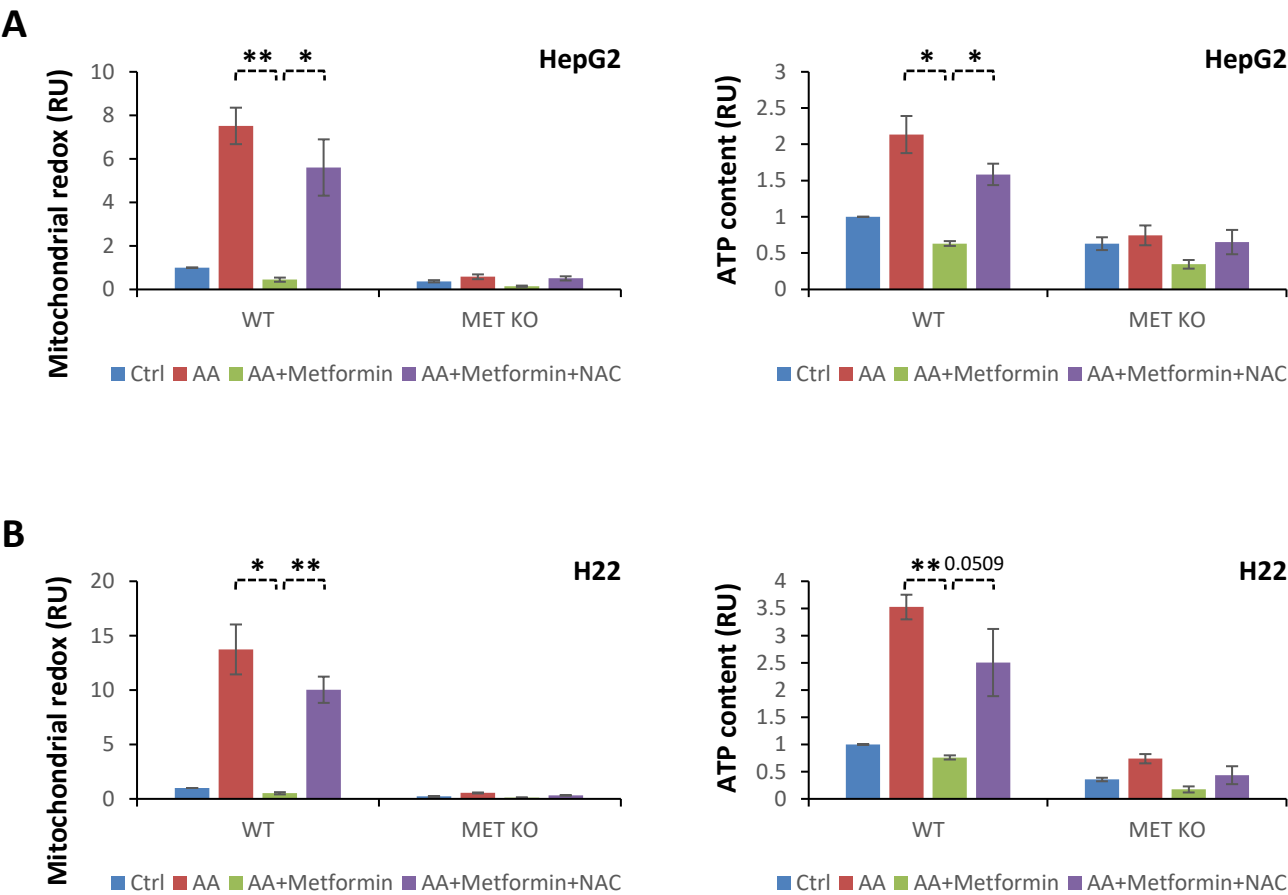

Figure S6



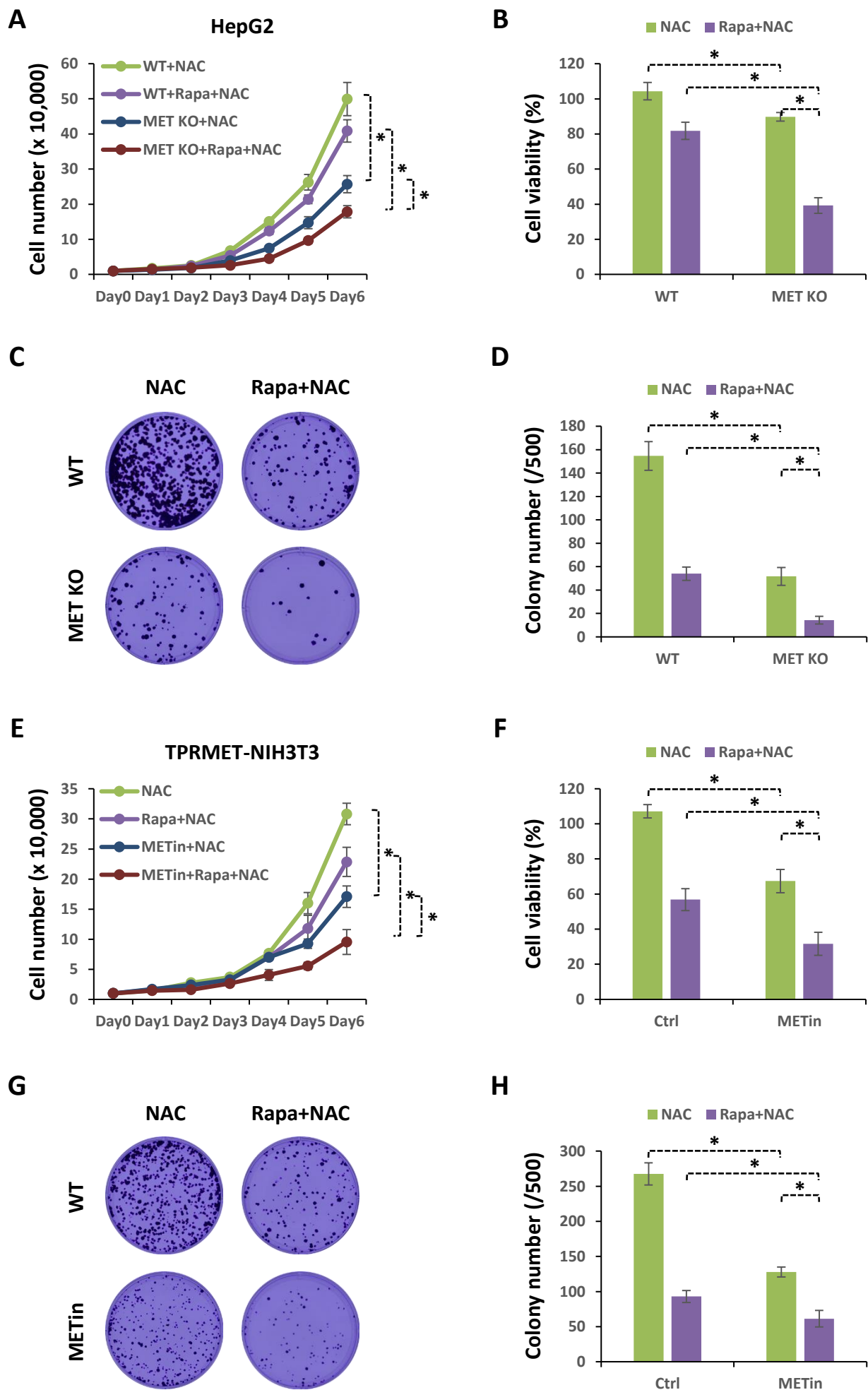

**Figure S8**

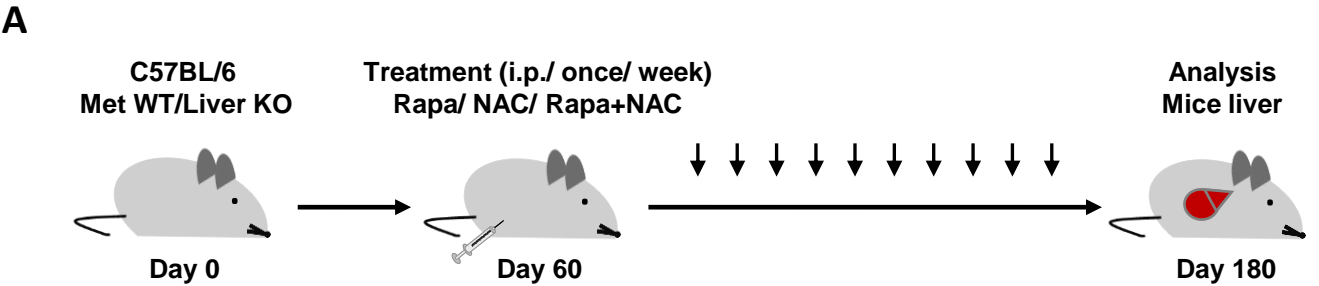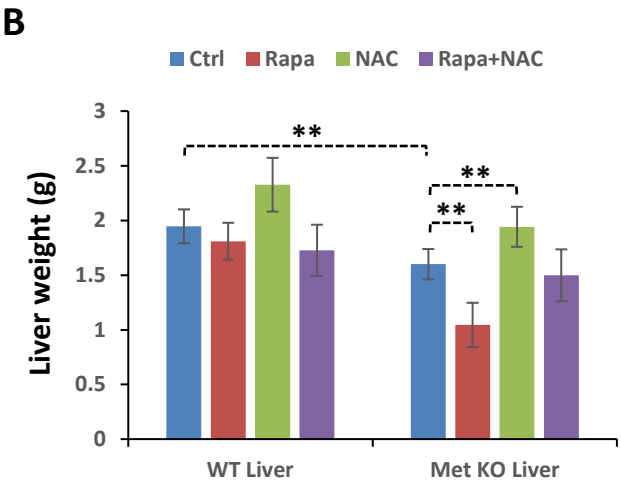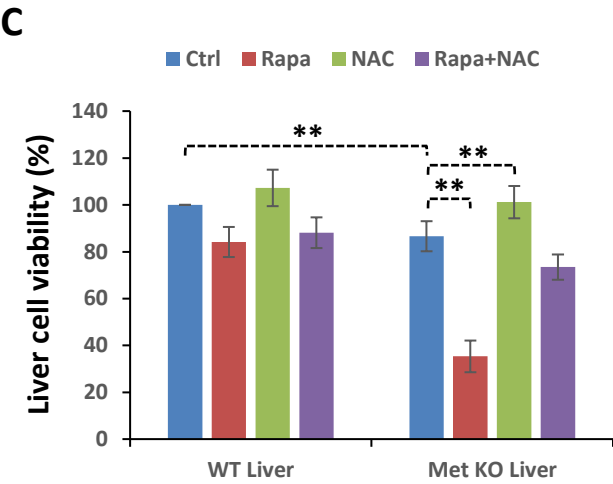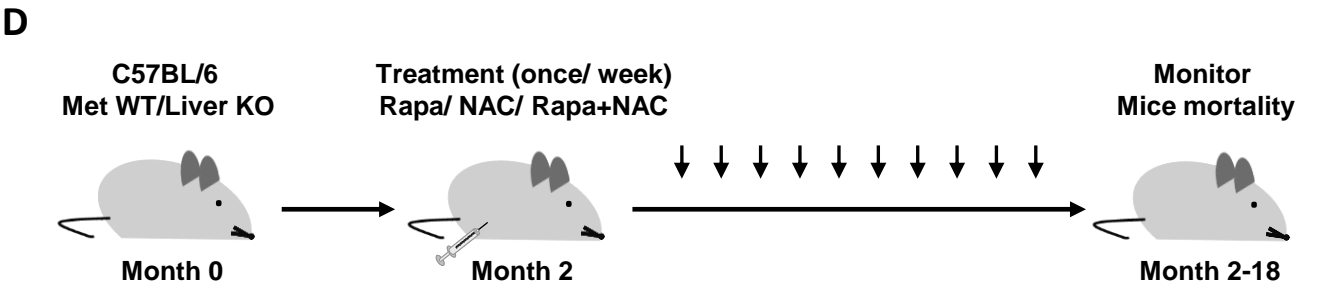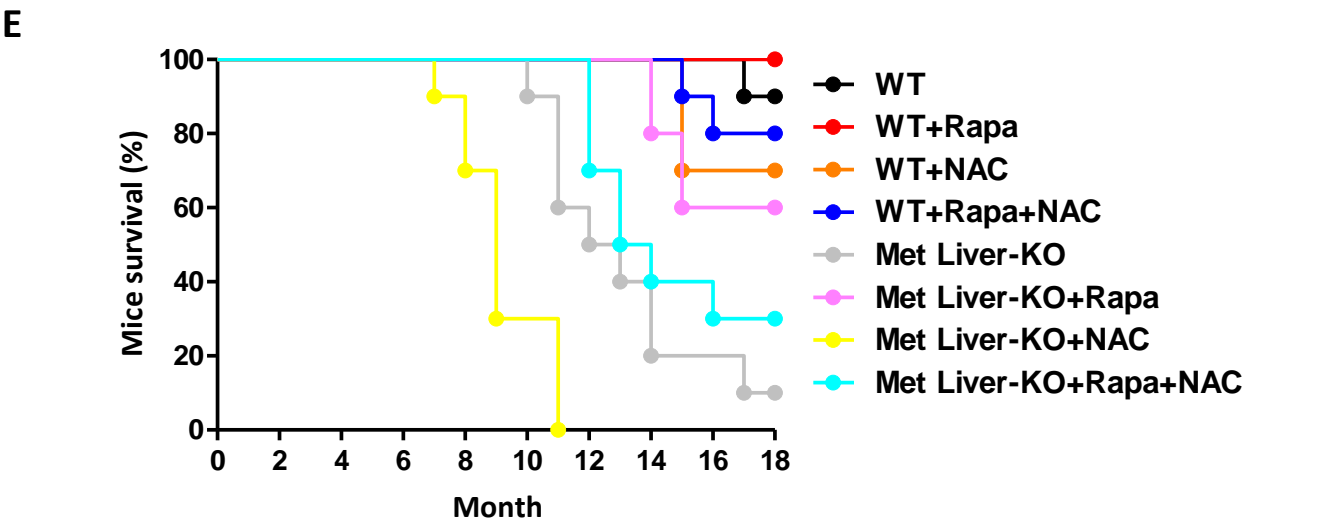

Figure S9

A

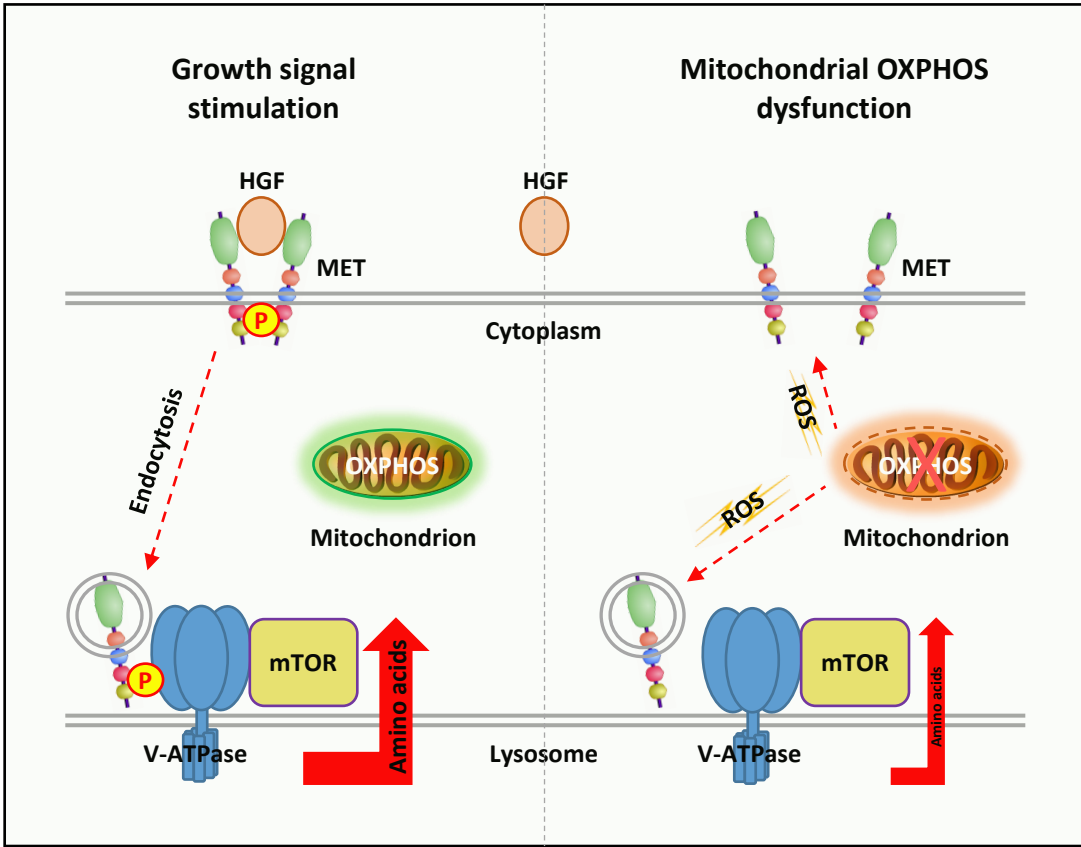

Figure S10

Supplement: Supplementary file 1 — Supporting Figure [file CTM2-10-e237-s001.pdf]
