# Supplementary material for: Combinational therapy targeting the MET‐mTOR‐ROS loop disrupts mitochondrial autoregulatory machinery of liver cancer
Source: Clin Transl Med. 2020 Dec 1;10(8):e237. doi: 10.1002/ctm2.237 (PMC7708774; doi:10.1002/ctm2.237)
Supplement: Supplementary file 2 — Supporting Figure [file CTM2-10-e237-s002.docx]

**Supplemental Figure Legends**

**Figure S1. MET controls AA-stimulated mTOR activation in a kinase-dependent manner.** (**A**) MET is required for AA-induced mTOR activation. WT and MET KO HepG2 cells (5 × 10^4^) were individually transfected with Flag-MET or vector control for 36 hours, then deprived of AA for 90 min, and subsequently stimulated with AA for 45 min. Cell lysates were subjected to immunoblot with indicated antibodies. (**B**) Capmatinib suppresses MET-mediated mTOR activation. MET KO HepG2 cells (5 × 10^4^) were individually transfected with Flag-MET or vector control for 36 hours, then deprived of AA for 90 min, and subsequently stimulated with AA for 45 min with or without 50 nM Capmatinib. Cell lysates were subjected to immunoblot with indicated antibodies. (**C**) MET-KD mutant loses the ability to mediate AA-stimulated mTOR activation. MET KO HepG2 cells (5 × 10^4^) were individually transfected with Flag-MET KD mutant or vector control for 36 hours, then deprived of AA for 90 min, and subsequently stimulated with or without AA for 45 min. Cell lysates were subjected to immunoblot with indicated antibodies. (**D**) TPR-MET fusion protein facilitates AA-stimulated mTOR activation. MET KO HepG2 cells (5 × 10^4^) were individually infected with TPR-MET or vesicle control for 48 hours, then deprived of AA for 90 min, and subsequently stimulated with or without AA for 45 min. Cell lysates were subjected to immunoblot with indicated antibodies. Blots are representative of at least 3 independent experiments.

**Figure S2. MET is essential for AA-stimulated mitochondrial OXPHOS.** (**A-B**) MET is essential for AA-stimulated mitochondrial redox *in vitro*. WT and MET KO HepG2 (A) or H22 (B) cells (5 × 10^5^) were starved for 90 min, and then stimulated with AA for 45 min and 90 min respectively, and subsequently subjected to mitochondrial redox analysis. (**C-D**) MET is essential for AA-stimulated ATP production *in vitro*. WT and MET KO HepG2 (C) or H22 (D) cells (5 × 10^5^) were starved for 90 min, and then stimulated with AA for 45 min and 90 min respectively, and subsequently subjected to ATP content analysis. (**E-F**) MET deficiency blocks AA-stimulated mitochondrial redox and ATP production in mouse. WT and Met^Liver-KO^ C57BL/6 mice were starved for one day, and then stimulated with AA for 90 min and 180 min respectively by intravenous injection (*i.v*.). Mice were subsequently sacrificed, dissected, and livers (1 g) were digested for hepatocyte cultures that were used to analyze mitochondrial redox (E) and ATP content (F). (**G-H**) HGF enhances AA-stimulated mitochondrial OXPHOS. WT and MET KO HepG2 cells (5 × 10^5^) were starved overnight, then stimulated with or without AA for 45 min and 90 min respectively in presence of 50 ng/ml HGF or not, and subsequently subjected to analysis of mitochondrial redox (G) and ATP content (H). (**I-J**) MET-KD mutant fails to mediate AA-stimulated mitochondrial redox and ATP production. MET KO HepG2 cells (5 × 10^5^) were individually transfected with Flag-MET, KD mutant and vector control for 36 hours, then deprived of AA for 90 min, and subsequently stimulated with or without AA for 45 min and 90 min respectively. After treatment, cells were subjected to analysis of mitochondrial redox (I) and ATP content (J). Data are presented as the means ± s.d. from at least 3 independent experiments, and statistically significant differences with two tailed Student’s t-test are marked as * (p < 0.05) or ** (p < 0.01).

**Figure S3. V-ATPase–mTOR axis is essential for MET-regulated AA-stimulated mitochondrial OXPHOS.** (**A**) MET regulates AA-stimulated mitochondrial redox via V-ATPase. HEK-293T cells (5 × 10^5^) were transfected with Flag-MET or vector control for 36 hours, then starved for 90 min in the presence of 5 μM Concanamycin A or not, and subsequently stimulated with or without AA for 45 min and 90 min respectively. After treatment, cells were subjected to mitochondrial redox analysis. (**B**) MET regulates AA-stimulated ATP production via V-ATPase. HEK-293T cells (5 × 10^5^) were treated as described above, and subsequently subjected to ATP content analysis. (**C**) MET regulates AA-stimulated mitochondrial redox via mTOR. HEK-293T cells (5 × 10^5^) were transfected with Flag-MET or vector control for 36 hours, then starved for 90 min in the presence of 50 nM Rapamycin or vehicle, and subsequently stimulated with or without AA for 45 min and 90 min respectively. After treatment, cells were subjected to mitochondrial redox analysis. (**D**) MET regulates AA-stimulated ATP production via mTOR. HEK-293T cells (5 × 10^5^) were treated as described above, and subsequently subjected to ATP content analysis. Data are presented as the means ± SD from at least 3 independent experiments, and statistically significant differences with two tailed Student’s t-test are marked as * (p < 0.05) or ** (p < 0.01).

**Figure S4. Mitochondrial OXPHOS feeds back to MET dimerization, phosphorylation state, V-ATPase binding and mTOR activating.** (**A-B**) Mitochondrial OXPHOS affects dimerization of MET. HEK-293T cells expressing Flag-MET (1 × 10^5^) were individually treated with or without 2 mM Metformin for 12 hours (A), or 2.5 μM Oligomycin plus 250 nM Antimycin A for 4 hours (B), then subjected to Native-PAGE and SDS-PAGE respectively, and subsequently analyzed by immunoblot with the indicated antibodies. (**C-D**) Mitochondrial OXPHOS determines the phosphorylation state of MET. HEK-293T cells expressing Flag-MET (5 × 10^4^) were individually treated with 2 mM Metformin for 12 hours (C), or 2.5 μM Oligomycin plus 250 nM Antimycin A for 4 hours (D). Cell lysates were subjected to immunoblot with indicated antibodies. (**E-F**) Mitochondrial OXPHOS dysfunction interrupts MET binding to V-ATPase complex. HEK-293T cells expressing Flag-MET (5 × 10^5^) were individually treated with Metformin (E), or Oligomycin plus Antimycin A (F) as described above. Cell lysates were subjected to immunoprecipitation and immunoblot with indicated antibodies. (**G-H**) Mitochondrial OXPHOS dysfunction interrupts MET-mediated mTOR activation. HEK-293T cells expressing Flag-MET (5 × 10^4^) were individually treated with Metformin (G), or Oligomycin plus Antimycin A (H) as described above, then deprived of AA for 90 min, and subsequently stimulated with AA for 45 min. Cell lysates were subjected to immunoblot with indicated antibodies. Blots are representative of at least 3 independent experiments.

**Figure S5. Mitochondrial OXPHOS dysfunction promotes a negative feedback by inactivation of MET–V-ATPase–mTOR axis.** (**A**) HGF stimulation aggravates cell death under mitochondrial OXPHOS dysfunction via MET, V-ATPase and mTOR. WT and MET KO HepG2 cells (5 × 10^4^) were starved overnight, and subsequently stimulated with or without 50 ng/ml HGF for 45 min, then treated with 2 mM Metformin for 12 hours in the presence of 5 μM Concanamycin A or 50 nM Rapamycin or vehicle. After treatment, cells were subjected to cell death analysis. (**B**) TPR-MET fusion protein aggravates cell death under mitochondrial OXPHOS dysfunction via V-ATPase and mTOR. WT and MET KO HepG2 cells (5 × 10^4^) were individually infected with TPR-MET or vesicle control for 48 hours, then treated with 2 mM Metformin for 12 hours in the presence of 5 μM Concanamycin A or 50 nM Rapamycin or vehicle. After treatment, cells were subjected to cell death analysis. (**C**) MET de-phosphorylation mediates a protective feedback to mitochondrial OXPHOS dysfunction. MET KO HepG2 cells (5 × 10^4^) were individually transfected with Flag-MET, KD mutant or vector control for 36 hours, then starved overnight, and subsequently stimulated with 50 ng/ml HGF for 45 min. After stimulation, cells were further treated with 2 mM Metformin for 12 hours in the presence of 10 nM Capmatinib or vehicle, and then subjected to cell death analysis. (**D**) MET de-phosphorylation prevents mitochondrial OXPHOS dysfunction-caused cell death via V-ATPase and mTOR. MET KO HepG2 cells (5 × 10^4^) were individually transfected with Flag-MET, KD mutant or vector control for 36 hours, then treated with 2 mM Metformin for 12 hours in the presence of 5 μM Concanamycin A or 50 nM Rapamycin or vehicle. After treatment, cells were subjected to cell death analysis. Data are presented as the means ± SD from at least 3 independent experiments, and statistically significant differences with two tailed Student’s t-test are marked as * (p < 0.05) or ** (p < 0.01).

**Figure S6. Mitochondrial ROS suspends AA-stimulated OXPHOS by targeting MET.** (**A-B**) Mitochondrial ROS suppresses AA-stimulated OXPHOS via MET. WT and MET KO HepG2 (A) or H22 (B) cells (5 × 10^5^) were individually treated with or without 2 mM Metformin for 12 hours or/and 5 mM NAC for 4 hours, then starved for 90 min and subsequently stimulated with AA for 45 min. After treatment, cells were subjected to mitochondrial redox and ATP content analysis. Data are presented as the means ± SD from at least 3 independent experiments, and statistically significant differences with two tailed Student’s t-test are marked as * (p < 0.05) or ** (p < 0.01).

**Figure S7. Autophagy is not involved in MET–mTOR–ROS loop.** (**A**) Autophagy deficiency has no impact on MET-controlled mTOR activation. WT and Atg5 KO mouse embryo fibroblast (MEF) cells (5 × 10^4^) were individually transfected with Flag-MET or vector control for 36 hours, then deprived of AA for 90 min, and subsequently stimulated with AA for 45 min. Cell lysates were subjected to immunoblot with indicated antibodies. (**B**) Auto-lysosomal degradation pathway is not involved in MET-controlled mTOR activation. MET KO HepG2 cells (5 × 10^4^) were individually transfected with Flag-MET or vector control for 36 hours, then deprived of AA for 90 min after pre-incubation with or without 50 nM Bafilomycin A1 for 2 hours, and subsequently stimulated with AA for 45 min. Cell lysates were subjected to immunoblot with indicated antibodies. (**C**) Autophagy is not involved in mitochondrial ROS–MET feedback-modulated mTOR activity. WT and Atg5 KO MEF cells (5 × 10^4^) were individually transfected with Flag-MET or vector control for 36 hours, then treated with or without 2 mM Metformin for 12 hours or/and 5 mM NAC for 4 hours. After treatment, cells were deprived of AA for 90 min, then stimulated with AA for 45 min, and subsequently subjected to immunoblot with indicated antibodies. (**D**) Autophagy is not involved in mitochondrial ROS–MET feedback-modulated OXPHOS. WT and Atg5 KO MEF cells (5 × 10^5^) were transfected and treated as described above, and subsequently subjected to mitochondrial redox and ATP content analysis. Blots are representative of at least 3 independent experiments. Data are presented as the means ± SD from at least 3 independent experiments, and statistically significant differences with two tailed Student’s t-test are marked as * (p < 0.05) or ** (p < 0.01).

**Figure S8.** **MET–mTOR–ROS loop contributes to cancer cell proliferation, viability and clonality *in vitro*.** (**A**) Impacts of MET–mTOR–ROS loop on cancer cell proliferation. WT and MET KO HepG2 cells (1 × 10^4^) were individually seeded overnight at day 0, and subsequently treated with 5 mM *N*-acetyl-l-cysteine (NAC) or/and 50 nM Rapamycin (Rapa) respectively. Growth medium was replaced every day. Cumulative cell numbers were counted at indicated times, and presented as growth curves. (**B**) Impacts of MET–mTOR–ROS loop on cancer cell viability. WT and MET KO HepG2 cells (2.5 × 10^3^) were individually seeded overnight, and subsequently treated with 5 mM NAC or/and 50 nM Rapa respectively for 8 hours. After that, cells were individually incubated with 0.25 mg ml^−1^ WST-8 solution at 37 °C for 2 hours. The absorbance at 450 nm was measured to calculate the percentage of viable cells. (**C-D**) Impacts of MET–mTOR–ROS loop on cancer cell colony formation. WT and MET KO HepG2 cells (0.5 × 10^3^) were individually seeded overnight, and subsequently treated with 5 mM NAC or/and 50 nM Rapa respectively for 2 weeks. Growth medium was replaced every 2 days. After that, cells were fixed with 4% cold PFA for 45 min, and then stained with 0.1% crystal violet for 2 hours at RT. Representative images were shown as indicated (C), and colony numbers were quantified under a microscopy (D). (**E**) Effects of MET–mTOR–ROS loop on cancer cell proliferation. HepG2 cells (1 × 10^4^) were individually seeded overnight at day 0, and subsequently treated with 10 nM Capmatinib (METin), 5 mM NAC, or/and 50 nM Rapa respectively, then subjected to cell proliferation analysis as described before. (**F**) Effects of MET–mTOR–ROS loop on cancer cell viability. HepG2 cells (2.5 × 10^3^) were individually seeded overnight, and subsequently treated with 10 nM METin, 5 mM NAC, or/and 50 nM Rapa respectively for 8 hours, then subjected to cell viability analysis as described before. (**G-H**) Effects of MET–mTOR–ROS loop on cancer cell colony formation. HepG2 cells (0.5 × 10^3^) were individually seeded overnight, and subsequently treated with 10 nM METin, 5 mM NAC, or/and 50 nM Rapa respectively for 2 weeks, then subjected to cell clonality analysis as described before. Data are presented as the means ± s.d. from at least 3 independent experiments, and statistically significant differences with two tailed Student’s *t*-test are marked as * (p < 0.05) or ** (p < 0.01).

**Figure S9. MET–mTOR–ROS loop controls liver development and** **mice lifespan.** (**A**) Strategy for evaluating physiological function of MET–mTOR–ROS loop on liver growth. (**B**) Impacts of MET–mTOR–ROS loop on mice liver weight. WT and Met Liver-KO C57BL/*6* mice (n=10) were individually treated with vehicle control (Ctrl), Rapamycin (Rapa, 5 mg/kg in 100 μl of PBS), or/and *N*-acetyl-l-cysteine (NAC, 50 mg/kg in 100 μl of PBS) respectively by *i.p.* injection every week from indicated time. After 180 days, all mice were sacrificed, and individual liver weights of each mouse were measured. (**C**) Impacts of MET–mTOR–ROS loop on primary liver cell viability. Individual liver samples (n=10) from each treated group in WT and Met Liver KO C57BL/*6* mice were individually dissected for primary hepatocytes, and then directly subjected to cell viability analysis. (**D**) Strategy for evaluating physiological function of MET–mTOR–ROS loop on lifespan of mice. (**E**) Influences of MET–mTOR–ROS loop on mice lifespan. WT and Met Liver-KO C57BL/*6* mice (n=20) were individually treated with vehicle control (Ctrl), Rapamycin (Rapa, 5 mg/kg in 100 μl of PBS), or/and *N*-acetyl-l-cysteine (NAC, 50 mg/kg in 100 μl of PBS) respectively by *i.p.* injection every week from indicated time. The mortality under physiological condition was regularly reported during 2-18 months according to the Kaplan-Meier method. Data are presented as the means ± s.d., and statistically significant differences with two tailed Student’s *t*-test are marked as * (p < 0.05) or ** (p < 0.01).

**Figure S10. Proposed model.** (**A**) A schematic model depicting that MET acts as a gate-keeper for maintaining homeostasis between AA-mediated mTOR activation and mitochondrial OXPHOS via lysosomal V-ATPase complex. HGF stimulates this process, whereas mitochondrial ROS propels a negative feedback by dephosphorylating MET.
