# Supplementary material for: Combinational therapy targeting the MET‐mTOR‐ROS loop disrupts mitochondrial autoregulatory machinery of liver cancer
Source: Clin Transl Med. 2020 Dec 1;10(8):e237. doi: 10.1002/ctm2.237 (PMC7708774; doi:10.1002/ctm2.237)
Supplement: Supplementary file 3 — Supporting Information [file CTM2-10-e237-s003.docx]

**Supplemental Materials and Methods**

**Antibodies, inhibitors, and other reagents:** Antibodies were obtained from the indicated sources: anti-MET (700261, Thermo Fisher, for WB; sc-161, Santa Cruz, for IP), anti-p-MET Y1234/1235 (#3077, Cell Signaling), anti-S6K1 (#9202, Cell Signaling), anti-p-T389-S6K1 (#9205, Cell Signaling), anti-ATP6V1A (17115-1-AP, Proteintech), anti-Flag (F1804-50UG, Sigma), anti-beta Actin (66009-1-Ig, Proteintech), anti-Rabbit IgG (HRP) (GTX221666-01, GeneTex), and anti-Mouse IgG (HRP) (GTX221667-01, GeneTex). Inhibitors were acquired from the designated suppliers: Antimycin A (A8674, Sigma), Bafilomycin A1 (1334, Tocris Bioscience), Capmatinib (S2788, Selleckchem), Concanamycin A (2656, Tocris Bioscience), Metformin (S1950, Selleckchem), NAC (S1623, Selleckchem), Oligomycin A (S1478, Selleckchem), and Rapamycin (S1039, Selleckchem). Plasmid: pBABE-puro TPR-MET was a gift from Bob Weinberg (Addgene plasmid # 10902). Other reagents were purchased from the indicated corporations: HGF (100-39, PeproTech), and Lipofectamine™ 3000 transfection reagent (L3000008, Invitrogen).

**Cell lines and cell culture:** HEK293T, HepG2, SMMC-7721, Huh-7, H22, Hepa1-6, and NIH-3T3 cell lines were individually obtained from Cell Bank of Chinese Academy of Sciences (Shanghai, China) or KeyGEN BioTECH (Nanjing, China), where mycoplasma contamination detection and short tandem repeat (STR) profiling were performed for quality and identity guarantee. MEF *Atg5* WT / KO cells were kindly provided by Quan Chen (Institute of Zoology, Chinese Academy of Sciences). All the cell lines and derived cells were maintained at 37 °C incubator with 5 % CO_2_ under standard conditions as specified by suppliers. Except for AA starvation as indicated, cells were respectively cultured in Dulbecco’s modified Eagle’s medium (DMEM) or Roswell Park Memorial Institute 1640 medium (RPMI-1640), supplemented with 2 mM L-glutamine, 1 % NEAA, 100 units/ml penicillin, 100 mg/ml streptomycin and 10 % FBS. No cell lines used in this work were commonly misidentified cell lines, according to the database from International Cell Line Authentication Committee (ICLAC). All the cell lines were freshly thawed from the purchased seed cells, cultured for no more than 2 months, and regularly checked by virtue of their morphological features to avoid cross-contamination or misuse.

**Generation of CRISPR/Cas9-mediated knockout cell lines and conditional knockout mice:** For generation of *MET* knockout cell lines, cells were individually co-transfected with MET CRISPR/Cas9 KO Plasmid (h) (sc-400101, Santa Cruz) and MET HDR Plasmid (h) (sc-400101-HDR, Santa Cruz); or Met CRISPR/Cas9 KO Plasmid (m) (sc-421635, Santa Cruz) together with Met HDR Plasmid (m) (sc-421635-HDR, Santa Cruz). MET/Met CRISPR/Cas9 KO Plasmid were designed to disrupt gene expression by causing a double-strand break (DSB) in a 5' constitutive exon within the *MET/Met* gene, and consisted of a pool of 3 plasmids, each encoding the Cas9 nuclease and a target-specific 20 nt guide RNA (gRNA) designed for maximum knockout efficiency. MET/Met HDR Plasmid also consisted of a pool of 2-3 plasmids, each containing a homology-directed DNA repair (HDR) template corresponding to the cut sites generated by the MET CRISPR/Cas9 KO Plasmid. Briefly, 1.5 × 10^5^ of cells were seeded in 3 ml of antibiotic-free standard growth medium per well in a 6-well tissue culture plate. When cell confluency up to 40 %, total 2 µg of plasmids were transfected into cells by UltraCruz® Transfection Reagent (sc-395739, Santa Cruz) according to the manufacturer's recommendation. 3 days later, 1 µg/ml puromycin (ant-pr-1, InvivoGen) was added into complete growth medium for at least 10 days of selection. After selection, cells were suspended, diluted and re-seeded to ensure single clone formation. Knockout efficiency in each single clone was evaluated by western blot with two different antibodies, and further verified by sequencing the genomic DNA. Met liver-conditional knockout mice (Met^Liver-KO^) were created by CRISPR/Cas9 system-mediated genome editing of *Met* gene in the C57BL/*6* mice at Nanjing Biomedical Research Institute of Nanjing University (Nanjing, China). In brief, *Met* gene had 22 exons, with the ATG start codon in exon3 and TGA stop codon in exon22. To generate Met knockout mice, exon3-targeted sgRNA and Cas9 mRNA were co-injected into zygotes where gRNA could direct Cas9 endonuclease to cleave *Met* gene and create a DSB, which would be repaired and resulted in a frameshift from exon4.

**Immunoprecipitation and immunoblot:** After rinsed three times with ice-cold PBS, cells were re-suspended in lysis buffer (25 mM HEPES, pH 7.5, 150 mM NaCl, 0.25 % Triton X-100, 0.25 % NP-40, 0.5 % CHAPS, 10 % glycerol, phosphatase inhibitor cocktail (B15001, Bimake) and protease inhibitor cocktail (B14001, Bimake)) on ice for 2 hours, and then centrifuged at 14,000 g for 20 min. The supernatants were pre-cleared with protein A/G-coupled agarose (sc-2003, Santa Cruz) for 2 hours, and subsequently subjected to incubation with 2.5 μg of the indicated antibodies or 25 μl anti-Flag gel (B23101, Bimake) overnight at 4 °C, followed by addition of 25 μl protein A/G agarose for another 3 hours to conjugate antibody complex. After washed four times with lysis buffer, immunoprecipitates were boiled in 1 × loading buffer for immunoblot analysis. Protein samples were further resolved by SDS-PAGE or Native-PAGE with 4-20 % 15-well ExpressPlus^TM^ PAGE Gel (M42015C, GenScript), and then transferred onto nitrocellulose membrane (66485, PALL). Membranes were blocked with 10 % BSA in TBST for 2 hours, and subsequently incubated with indicated primary antibodies according to manufacturer’s recommendation. After washed with TBST for three times, membranes were incubated with appropriate HRP-labeled secondary antibodies. Immunolabeling was developed with SuperSignal™ West Femto Maximum Sensitivity Substrate (34095, Thermo Fisher). Visualized images were obtained using ImageQuant^TM^ LAS-4000 (GE Fujifilm) or photographic film. The similar settings for exposure time, brightness, contrast and scanning condition were applied to capture parallel digital images.

**Mitochondrial OXPHOS analysis:** To analyze mitochondrial OXPHOS, cells were cultured and treated as indicated, and subsequently subjected to detection of mitochondrial oxidation-reduction reaction and ATP production respectively. Specific assay kits were used according to the manufacturer’s instructions, and listed as follows: Mitochondrial Viability Stain Kit (ab129732, Abcam) and ATP Assay Kit (MAK190, Sigma). The viability of mitochondrial redox and the content of ATP were individually measured and quantified using iMarkTM Microplate Reader (#168-1130, Bio-Rad), and then normalized to the relevant protein concentration.

**Cell proliferation, viability and clonality analysis:** To analyze cellular proliferation, 1 × 10^4^ of each indicated cells were seeded in 6-well tissue culture plates at day 0 in triplicates in 3 ml of normal growth medium per well. The medium was changed every day. Cell number at the indicated time points was counted after Trypsin digestion using a haemocytometer, and recorded for analysis. For cell viability assay, 2.5 × 10^3^ of each indicated cells were seeded in 96-well microplates in triplicates in 100 μl of normal growth medium per well. After 48 hours, cells were further incubated with 0.25 mg/ml WST-8 solution at 37 °C for 2 hours, followed by addition of 10 μl of 3% SDS to end the reaction. The absorbance at 450 nm was measured, and the percentage of viable cells was calculated and averaged for each well. For clonality analysis, 500 of each indicated cells were seeded in 6-well plates at day 0 in triplicates in 3 ml of regular growth media supplemented with 20% FBS per well. Cells were maintained at 37 °C under 5% CO_2_ for 2 weeks, and growth medium was replaced every 2 days. Remaining cells were fixed with 4% cold paraformaldehyde (PFA) for 45 min, and then stained with 0.1% (w/v) crystal violet (SSI1047-1, SunShineBio), dissolved in 10% methanol, for 2 hours at RT. After extensive wash with distilled water until the background becoming clear, images of each replicate were photographed using a digital camera, and colony numbers were quantified under a microscopy.

**Animal use and care:** All animal research in this study were approved and supervised by the Institutional Animal Care and Use Committee (IACUC) of Institute of Life Sciences (ILS) at Southeast University (SEU). All animal experiments strictly adhered to protocols, policies and ethical guidelines formulated by our IACUC. Athymic *nu*/*nu* mice were obtained from the Comparative Medicine Center, Yangzhou University (Yangzhou, China); C57BL/*6* mice were obtained from the Nanjing Biomedical Research Institute of Nanjing University (Nanjing, China). All mice were housed in specific pathogen-free (SPF) conditions in the animal facility of School of Medicine at SEU, maintained at constant temperature and humidity under 12 h light-dark cycles, and received food and water *ad libitum*. After 2-3 weeks of adaptive phase, these mice were used in relative studies. None of the burden tumors exceeded the limit of neoplastic lesions (20% of mouse body mass or 20mm in longest axis) for humane care consideration.

**Tumor combined therapy:** To evaluate synergistic therapeutic efficiency of MET inhibition and mTOR blockage and/or ROS elimination, 1 × 10^6^ equal numbers of human-derived SMMC-7721 and Huh-7 liver cancer cells, or 5 × 10^6^ human TPRMET-driven mouse NIH3T3 cells were individually prepared in 100 μl of PBS, and then inoculated subcutaneously (*s.c.*) into the right flank of athymic *nu*/*nu* mice (male, 6 week-old) respectively. After inoculation, mice were monitored daily and weighed twice weekly. When tumors became visible, tumor growth was routinely measured by caliper, and reported as tumor surface size (longest dimension × perpendicular dimension). Once tumor reached 37.5-42.5 mm^2^, mice received PBS (vehicle control, 100 μl), MET inhibitor Capmatinib (METin, 20 mg/kg in 100 μl of PBS), mTOR blocker Rapamycin (Rapa, 10 mg/kg in 100 μl of PBS), or/and ROS scavenger *N*-acetyl-l-cysteine (NAC, 100 mg/kg in 100 μl of PBS) respectively, by subcutaneous multi-point injection adjacent to tumors every 5 days after caliper measurement. Mice were euthanatized at indicated times or when tumors reached maximum permitted condition for the ethical consideration. After sacrifice, bearing-tumors were dissected and weighed for further analysis. All experimental groups contained 5 mice and were run at least twice. Each one of the total 10 mice under same treatment showed same tendency, and were analyzed together.

**Mice survival analysis:** To assess the protective role of targeting MET-mTOR-ROS loop in caner survival, 5 × 10^5^ equal numbers of mouse-derived H22 and Hepa1-6 liver cancer cells were individually inoculated intraperitoneally (*i.p.*) into athymic *nu*/*nu* mice (male, 6 week-old). After 10 days, mice were treated with PBS (Ctrl, 100 μl), Capmatinib (METin, 30 mg/kg in 100 μl of PBS), Rapamycin (Rapa, 15 mg/kg in 100 μl of PBS), or/and *N*-acetyl-l-cysteine (NAC, 150 mg/kg in 100 μl of PBS) respectively by *i.p.* injection every 5 days. The mortality was regularly reported with 10 mice per group during 2 months according to the Kaplan-Meier method. To investigate the potential impact of MET-mTOR-ROS loop on mice lifespan under physiological condition, WT and Met Liver-KO C57BL/*6* mice (male, 2 month-old) were individually treated with or without Rapamycin (Rapa, 5 mg/kg in 100 μl of PBS) or/and *N*-acetyl-l-cysteine (NAC, 50 mg/kg in 100 μl of PBS) by *i.p.* injection every week. The mortality was regularly reported with 20 mice per group according to the Kaplan-Meier method, and 10 mice were sacrificed at day 180 for liver investigation.

**Statistical analyses:** Statistical analysis was performed with Excel 2015 (Microsoft Corporation) or GraphPad Prism 5 (GraphPad Software, Inc.) software to assess the differences between experimental groups. All images of western blot are representative of at least three independent experiments. All mice studies contain five individuals for each group and have been independently performed twice, and each one of the total ten mice under same treatment yields similar results. The variability within each group has been quantified from at least triplicate technical or biological replicates, and presented as means ± s.d. respectively. Statistical significance was determined by means of the two-tailed paired or unpaired Student’s t-test, and expressed as a p-value. * p-value < 0.05, ** p-value < 0.01, as compared to the negative, untreated or scrambled control. p-value < 0.05 was considered statistically significant.
